# Segmental fECV measurement using PCD-CT as an early predictor of liver function decline in chronic liver disease

**DOI:** 10.1007/s00261-025-05307-x

**Published:** 2025-11-24

**Authors:** Takashi Ohtani, Masato Shimada, Kouki Takahashi, Sayuri Hakoda, Yukihiro Tomita, Kiyotaka Takeuchi, Satomi Kanai, Tasuku Wakabayashi, Ayaki Kitano, Kenji Takata, Toshiki Tateishi, Tetsuya Tsujikawa

**Affiliations:** 1 Radiological Center, University of Fukui Hospital, Fukui, Japan; 2 Department of Radiology, University of Fukui, Fukui, Japan

**Keywords:** fECV, PCD-CT, CLD

## Abstract

**Purpose:**

The aim of this study was to investigate the utility of segmental hepatic extracellular volume fractions (fECV) calculated using a photon-counting detector CT (PCD-CT) for predicting liver function decline, as reflected by the modified albumin-bilirubin (mALBI) grade in chronic liver disease (CLD).

**Materials and methods:**

A total of 146 patients with CLD underwent PCD-CT of the liver, including 3-minute equilibrium phase imaging. Iodine densities of the aorta and liver segments (S2-S8), based on Couinaud’s classification, were measured to calculate fECV. The diagnostic accuracy of segmental fECV for predicting early liver function decline was assessed using mALBI grade as the functional reference standard, with receiver operating characteristic (ROC) analysis.

**Results:**

For mALBI grades 1, 2b, and 3, no significant differences in fECV were found among segments. For mALBI grade 2a, fECV values were significantly larger in S4 and S5 compared to S2, S3, S6, and S7 (*p* < 0.05), and the fECV in S8 was significantly larger than in S7 (*p* < 0.05). The fECV values in S6 and S7 significantly increased with higher mALBI grade, while other segments showed less pronounced increases. ROC analysis revealed that fECV in S4, S5, and S8 demonstrated sensitivities, specificities, and accuracies of approximately 90% for predicting early liver function decline (distinguishing mALBI grade 1 from grades 2a or above).

**Conclusion:**

Segmental fECV measurement using PCD-CT offers preliminary insights into liver function. Increased fECV in the medial (S4) and anterior (S5, S8) segments showed potential as an early indicator of functional decline, while fECV in the posterior segments (S6, S7) suggested utility in disease staging. As a proof-of-concept study, these findings require further validation before this approach can be adopted in clinical practice.

## Introduction

Chronic liver disease (CLD) is a major global health issue, with liver fibrosis being a key determinant of prognosis. Liver biopsy, despite being the gold standard for assessing fibrosis, has significant limitations, including its invasive nature, potential for sampling errors, and interobserver variability [1–3]. These drawbacks underscore the urgent need for reliable, non-invasive methods for the quantitative assessment of liver fibrosis and function.

While magnetic resonance elastography (MRE) is a well-established and accurate method for staging liver fibrosis, the hepatic extracellular volume fraction (fECV), which reflects the expansion of the extracellular matrix due to fibrosis, has also emerged as a promising non-invasive imaging biomarker. Computed tomography (CT) can be used to measure fECV by quantifying iodine concentration in the liver parenchyma and blood pool at equilibrium [4–9]. Recently, photon-counting detector CT (PCD-CT) has advanced this technique. With its superior energy resolution and material decomposition capabilities, PCD-CT allows for more accurate and precise quantification of iodine density compared to conventional energy-integrating detector CT (EID-CT), thereby improving the accuracy of fECV measurements [10–12].

Notably, the progression of liver fibrosis and subsequent functional decline is often a spatially heterogeneous process. This heterogeneity is largely attributed to the liver’s dual blood supply from the portal vein and hepatic artery, leading to regional variations in perfusion and susceptibility to injury across different liver segments [13, 14]. Consequently, assessing the liver with a single, whole-organ average value may obscure early, localized changes and underestimate the true extent of the disease. We hypothesized that a segmental analysis could provide a more sensitive measure of early functional deterioration.

Therefore, the purpose of this study was to investigate the utility of segmental fECV measured with PCD-CT for predicting early liver function decline in patients with CLD.

## Materials and methods

### Study population

**Fig. 1:**
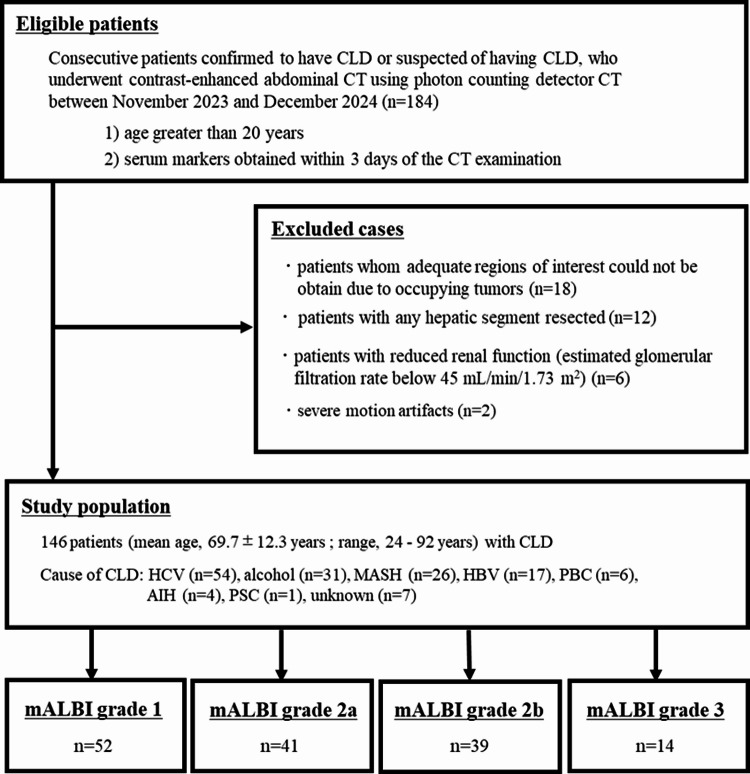
Flowchart of the study population. AIH = autoimmune hepatitis, ALBI = albumin–bilirubin, CLD = chronic liver disease, HBV = hepatitis B virus, HCV = hepatitis C virus, MASH = metabolic dysfunction associated steatohepatitis, PBC = primary biliary cholangitis, PSC = primary sclerosing cholangitis

This single-center retrospective study was approved by our institutional review board (reference no. 20230102), and the requirement for individual informed consent was waived for this retrospective analysis. This study included consecutive patients with either confirmed or suspected CLD who underwent contrast-enhanced abdominal CT using PCD-CT between November 2023 and December 2024. Inclusion criteria were: (1) age > 20 years; and (2) serum markers obtained within 3 days of the CT examination. Exclusion criteria were: patients in whom adequate regions of interest (ROIs) could not be placed due to space-occupying tumors (*n* = 18); patients with prior hepatic segment resection (*n* = 12); patients with reduced renal function (estimated glomerular filtration rate below 45 mL/min/1.73 m²) (*n* = 6); and patients with severe motion artifacts (*n* = 2). Finally, 146 patients (92 men and 54 women) with CLD were included in this study (Fig. 1). The cohort included patients confirmed to have CLD by liver biopsy (*n* = 67) and patients diagnosed with CLD by gastroenterologists based on various clinical findings (*n* = 79). Clinical findings included hepatitis virus infection, alcoholism, characteristic CT findings (splenomegaly, ascites, morphological changes of the liver, varices), and laboratory data suggestive of CLD.

Patients were classified according to the modified albumin-bilirubin (mALBI) grade based on the ALBI score: (log10 bilirubin [µmol/L] × 0.66) + (albumin [g/L] × −0.0852). The mALBI grade was assigned as follows: ALBI score ≤ − 2.60 for mALBI grade 1; ALBI score > − 2.60 to < − 2.27 for mALBI grade 2a; ALBI score ≥ − 2.27 to ≤ − 1.39 for mALBI grade 2b; and ALBI score > − 1.39 for mALBI grade 3. This simple and objective method effectively classifies liver function to estimate patient prognosis in CLD [15–17].

### CT examination/imaging technique

All CT examinations were performed using a PCD-CT system (NAEOTOM Alpha; Siemens Healthcare, Forchheim, Germany). An iodine contrast agent (500 mgI/kg) was administered intravenously over 30 s using an injector. Equilibrium phase images were acquired 3 min after the start of contrast injection. This timing reflects the standard protocol routinely used in our institution’s clinical liver dynamic CT examinations and has also been reported in previous studies to be useful for fECV evaluation. Imaging parameters were: detector collimation, 0.4 mm × 144; tube voltage, 120 kV; CARE keV IQ level, 180; gantry rotation speed, 0.5 s; pitch, 0.8; matrix, 512 × 512; field-of-view, 300–500 mm; reconstruction section thickness, 3 mm; and reconstruction interval, 3 mm. The average CT dose index volume (CTDIvol) in the equilibrium phase was 8.42 ± 2.38 mGy.

### Identification of hepatic segmentation

Liver segments were classified according to Couinaud’s classification into eight segments; S2 through S8 were evaluated [18]. S2 and S3 constitute the lateral segment of the left hepatic lobe, identified relative to the left hepatic vein and measured at the portal umbilical level. S4 is the medial segment of the left hepatic lobe, identified relative to the middle hepatic vein. S5 and S8 are the anterior segments of the right hepatic lobe, while S6 and S7 are the posterior segments of the right hepatic lobe, identified relative to the middle and right hepatic veins. Segment S1 (caudate lobe) contains numerous small blood vessels near the origin of the hepatic vein. Consequently, iodine density values in S1 are thought to be potentially overestimated due to partial volume effects from these vessels. Unlike other segments that drain into the inferior vena cava via the major hepatic veins (left, middle, and right), the caudate lobe drains through several independent small hepatic veins [19, 20]. Therefore, S1 was excluded from fECV measurements in this study. Given that S1 constitutes a very small fraction of the total liver volume, its exclusion is considered to have a negligible impact on the overall interpretation of whole-liver fECV.

### Imaging analysis

Iodine density maps were generated from the equilibrium phase images on the CT console. Subsequent analysis was performed on a workstation (Syngo.via version VB30A; Siemens Healthineers, Germany). First, a 10-mm² circular ROI was placed within the abdominal aorta, carefully avoiding calcifications and the aortic wall, to measure the iodine density of the aorta. Next, three 10-mm² circular ROIs were placed within each liver segment (S2 – S8), avoiding focal lesions, large vessels, and the liver capsule. ROIs were placed in areas that appeared visually homogeneous and in the relatively central region of each segment to minimize partial volume effects. The placement strategy followed methods established in previous studies [4] to enhance methodological consistency. Three ROIs were selected at different locations within these constraints to ensure reproducibility, and their average iodine density value was used for each segment. The fECV was calculated using the following formula:

$$ \begin{aligned} fECV\left( \% \right) = & \left( {1 - Hematocrit} \right) \\ & \times \left( {Iodine\,density\,of\,liver/Iodine\,density\,of\,aorta} \right) \\ & \times 100 \\ \end{aligned} $$

**Fig. 2:**
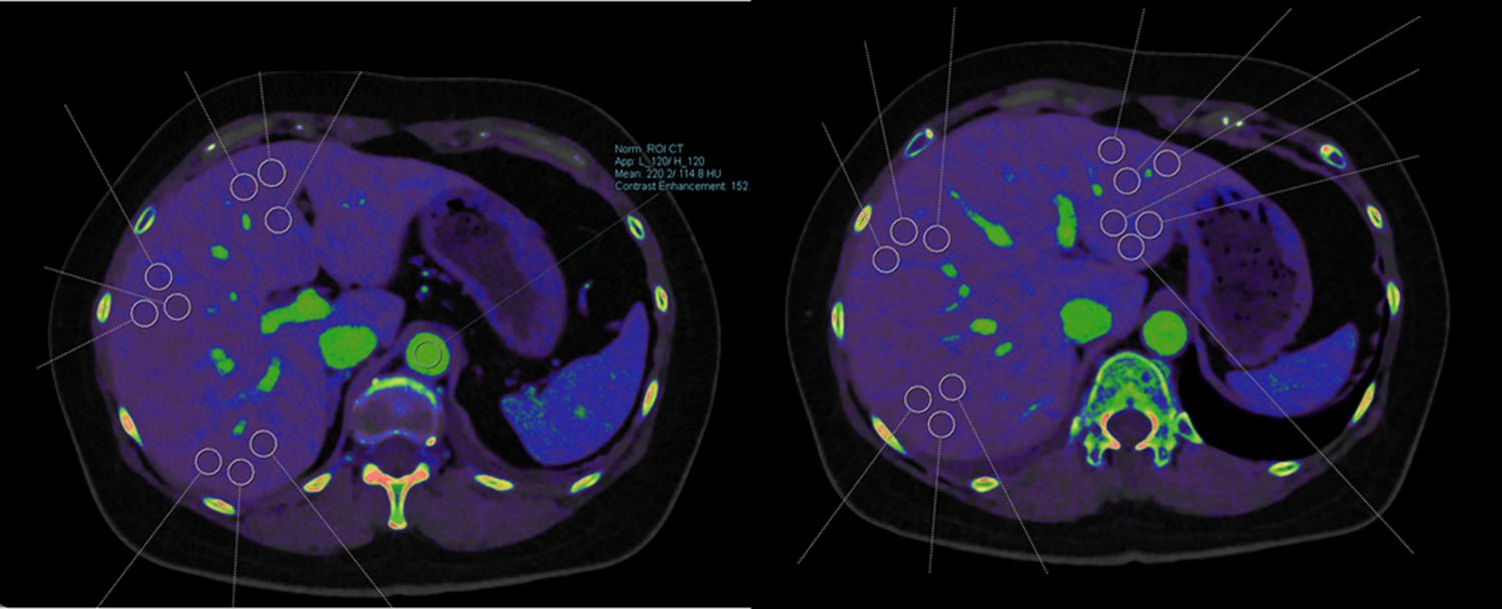
Example of iodine density measurement on an axial PCD-CT image. Iodine density was measured in seven segments (S2–S8) according to Couinaud’s classification. Three 10-mm² circular ROIs were placed within each segment, avoiding focal lesions and large vessels. The average density from these three ROIs represented the segmental iodine density. An ROI was also placed in the aorta

Two radiological technologists, who underwent a dedicated training session on the study protocol, independently performed all ROI placements and subsequent fECV calculations. To ensure the accuracy and consistency of these measurements, a board-certified radiologist with 15 years of experience in abdominal imaging, who was not involved in the initial measurements, reviewed every ROI placement. If an ROI was judged to be inappropriately placed (e.g., overlapping a major vessel and a lesion, or crossing a segmental boundary), the measurement was invalidated, and the technologist was required to perform a new measurement for that segment. The final fECV value used for analysis was the average of the two independent, radiologist-approved measurements. An example of appropriate ROI placement is shown in Fig. 2.

### Statistical analyses

The sex distribution among mALBI grades was compared using the chi-square test. Body mass index, age, red blood cell, hemoglobin, hematocrit, albumin, and total bilirubin were compared among grades using the Kruskal-Wallis test. The Shapiro-Wilk test was used to assess the normality of fECV distributions. Interobserver agreement for fECV measurements between the two radiological technologists was evaluated using intraclass correlation coefficients (ICCs; two-way mixed model). ICCs were interpreted as follows: < 0.50, poor; 0.50–0.74, moderate; 0.75–0.89, good; and 0.90–1.00, excellent [21].

Spearman’s rank correlation coefficient was used to evaluate the correlation between segmental fECV and mALBI grade. The fECV of each liver segment was compared among mALBI grades using one-way analysis of variance (ANOVA) with the post hoc Holm-Bonferroni test. Receiver operating characteristic (ROC) analysis was performed to evaluate the diagnostic accuracy of segmental fECV for predicting early liver function decline (defined as distinguishing mALBI grade 1 from grades 2a or above) and to determine optimal cutoff values. The area under the ROC curve (AUC) and its 95% confidence interval (CI) were calculated. The optimal cutoff value was determined using the Youden index method. Sensitivity, specificity, positive predictive value (PPV), negative predictive value (NPV), and accuracy were also calculated. All statistical analyses were performed using SPSS statistical software (version 21.0; IBM Corp., Armonk, NY, USA).

## Results

### Study population

Patient clinical characteristics are summarized in Table 1. No significant differences in sex ratio (*p* = 0.373), age (*p* = 0.160), or body mass index (*p* = 0.789) were observed among the four groups; however, red blood cell, hemoglobin, and hematocrit significantly decreased as liver function declined (*p* < 0.001).

**Table 1:** Clinical features of the patients in this study

	mALBI grade				*p* value
	1	2a	2b	3	
No. of participants	52	41	39	14	
Gender (Men/ Women)	35/17	27/14	20/19	10/4	0.373
Age (years)	67.7 ± 11.8	72.4 ± 12.3	70.1 ± 12.1	67.6 ± 12.3	0.160
Body mass index (kg/m^2^)	23.3 ± 5.5	23.2 ± 3.1	22.5 ± 4.3	23.4 ± 5.6	0.789
Red Blood Cell (10^6^/µL)	4.39 ± 0.60	3.89 ± 0.61	3.64 ± 0.62	3.54 ± 0.79	< 0.001
Hemoglobin (g/dL)	13.2 ± 1.7	12.1 ± 1.8	11.1 ± 1.9	12.2 ± 2.1	< 0.001
Hematocrit (%)	41.1 ± 4.5	37.7 ± 4.4	34.0 ± 5.1	34.2 ± 6.0	< 0.001
Albumin (g/dL)	4.38 ± 0.28	3.71 ± 0.19	3.27 ± 0.32	2.49 ± 0.25	< 0.001
Total bilirubin (mg/dL)	0.838 ± 0.42	0.763 ± 0.48	1.58 ± 1.8	2.93 ± 2.58	< 0.001
ALBI score	-2.98 ± 0.24	-2.46 ± 0.10	-1.95 ± 0.22	-1.10 ± 0.28	< 0.001

Continuous variables are expressed as mean ± standard deviation. Categorical variables are expressed as numbers. ALBI = albumin-bilirubin, mALBI = modified albumin-bilirubin

### Regional fECV and mALBI grade

For all mALBI grades, fECV values were normally distributed (*p* ≥ 0.05). The fECV for each mALBI grade in each liver segment is shown in Table 2. Interobserver agreement for fECV measurements was good to excellent (ICC range: 0.809–0.940). The correlation coefficients between mALBI grade and fECV in each liver segment were as follows: S2, 0.706; S3, 0.725; S4, 0.722; S5, 0.738; S6, 0.761; S7, 0.756; S8, 0.725 (all *p* < 0.001, Spearman’s rank correlation).

**Table 2:** Hepatic fECV of each segment by mALBI grade, and interobserver agreement

fECV	mALBI grade			
	1	2a	2b	3
S2	17.7 ± 3.82	22.1 ± 3.88	26.0 ± 5.03	31.1 ± 7.37
ICC (95% CI)	0.901 (0.834–0.941)	0.898 (0.815–0.944)	0.881 (0.786–0.936)	0.877 (0.659–0.959)
S3	17.2 ± 3.32	22.0 ± 3.70	25.8 ± 5.06	30.2 ± 7.37
ICC (95% CI)	0.826 (0.717–0.896)	0.891 (0.804–0.941)	0.849 (0.732–0.918)	0.862 (0.635–0.953)
S4	17.2 ± 3.45	26.0 ± 3.46	26.2 ± 4.56	30.8 ± 6.21
ICC (95% CI)	0.874 (0.791–0.925)	0.891 (0.797–0.942)	0.834 (0.706–0.909)	0.922 (0.782–0.974)
S5	17.4 ± 3.76	25.3 ± 3.97	26.1 ± 4.13	31.7 ± 6.18
ICC (95% CI)	0.924 (0.873–0.956)	0.844 (0.725–0.914)	0.911 (0.838–0.952)	0.909 (0.744–0.970)
S6	17.7 ± 3.47	22.0 ± 3.91	26.1 ± 3.67	31.8 ± 6.04
ICC (95% CI)	0.913 (0.854–0.949)	0.877 (0.767–0.935)	0.814 (0.639–0.903)	0.940 (0.824–0.980)
S7	17.3 ± 3.21	21.7 ± 4.12	26.0 ± 4.31	31.3 ± 6.19
ICC (95% CI)	0.844 (0.744–0.907)	0.923 (0.859–0.958)	0.828 (0.694–0.906)	0.889 (0.692–0.963)
S8	17.2 ± 3.52	24.3 ± 4.29	25.7 ± 4.49	31.3 ± 6.50
ICC (95% CI)	0.898 (0.830–0.940)	0.857 (0.746–0.922)	0.820 (0.684–0.901)	0.869 (0.638–0.956)

Continuous variables are expressed as mean ± standard deviation. CI = confidence interval, ICC = intraclass correlation coefficient

**Fig. 3:**
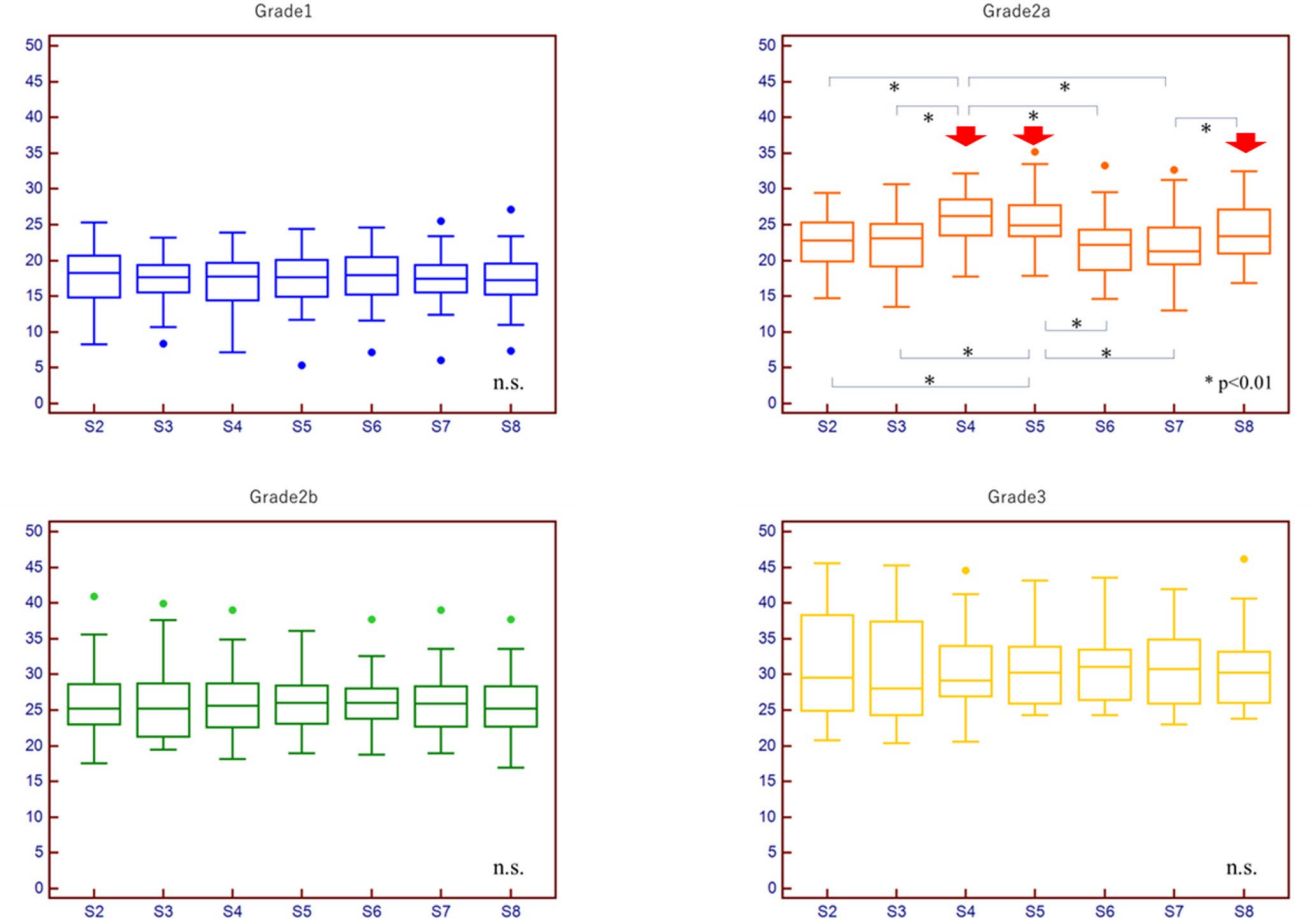
Box plots comparing hepatic fECV among liver segments (S2–S8) stratified by mALBI grade. No significant difference in fECV was found among segments in mALBI grades 1, 2b, and 3. In mALBI grade 2a (B), fECVs were significantly larger in S4 and S5 compared to S2, S3, S6, and S7, and fECV was significantly larger in S8 than in S7 (*p* < 0.05). Arrows indicate the segments with statistically significant fECV elevation. Whiskers indicate the range, boxes the interquartile range, and lines the median

In mALBI grades 1, 2b, and 3, no significant difference in fECV was found among segments (Fig. 3A, C and D). In mALBI grade 2a, fECV values were significantly larger in S4 and S5 than in S2, S3, S6, and S7 (*p* < 0.05), and the fECV in S8 was significantly larger than in S7 (*p* < 0.05) (Fig. 3B).

**Fig. 4:**
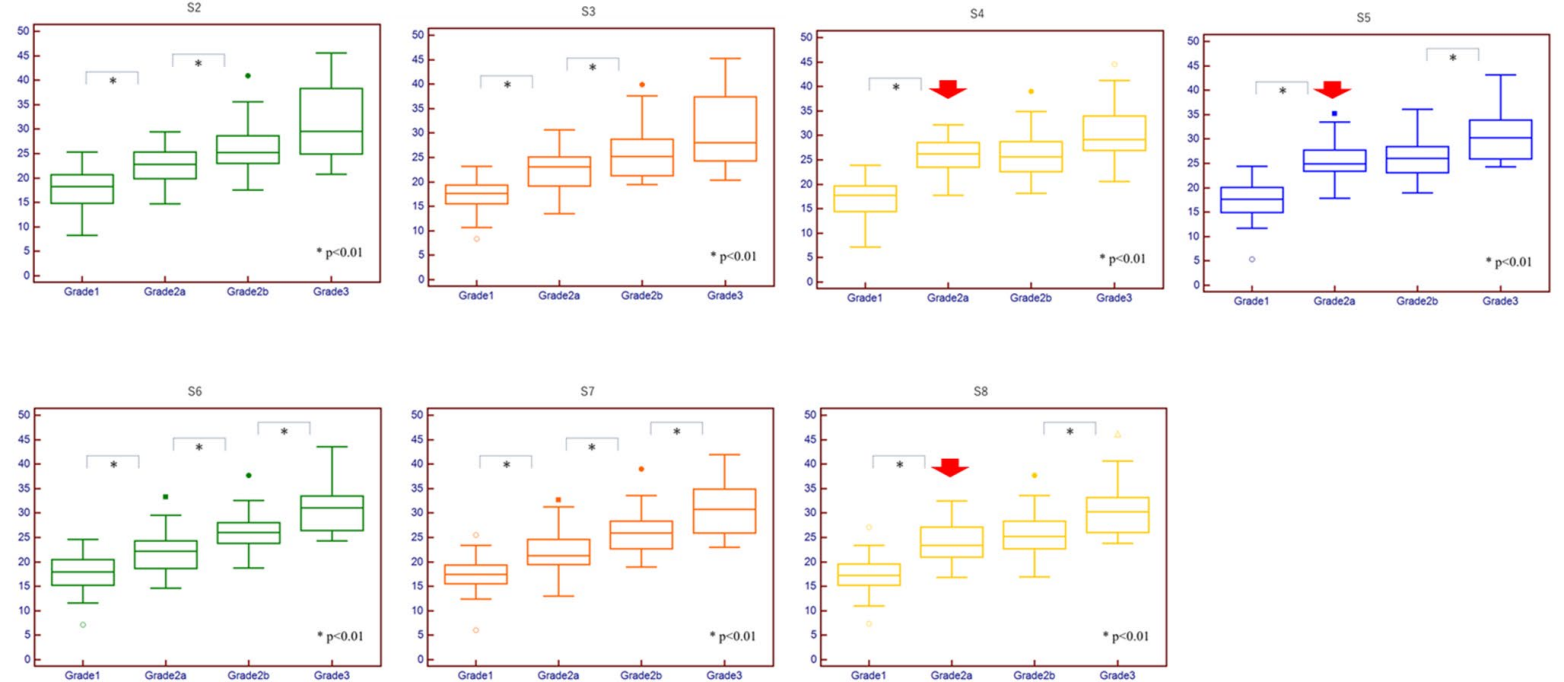
Box plots showing hepatic fECV for each segment (S2-S8) across different mALBI grades. Significant differences were observed between all consecutive grades in S6 and S7 (*p* < 0.01). However, no significant differences were observed between grades 2b and 3 in S2, S3, and S4, nor between grades 2a and 2b in S4, S5, and S8 (arrows). Whiskers indicate the range, boxes the interquartile range, and lines the median

In all liver segments, a trend of increasing fECV with higher mALBI grade was observed. In S6 and S7, significant differences in fECV were observed between all consecutive grades (*p* < 0.01). However, in S2, S3, and S4, no significant differences were observed between grades 2b and 3. Similarly, in S4, S5, and S8, no significant differences were observed between grades 2a and 2b (Fig. 4A-G).

**Fig. 5:**
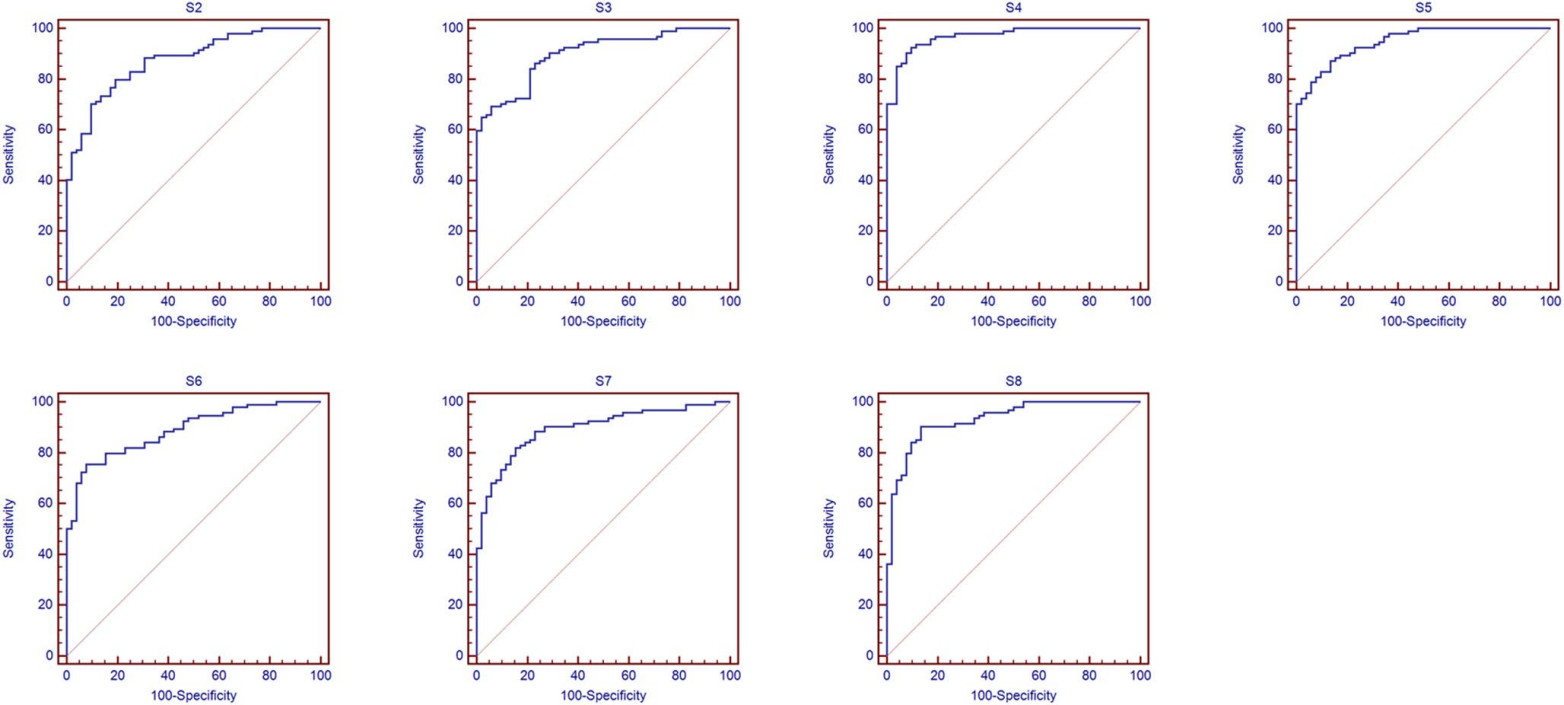
ROC curves demonstrating the ability of segmental fECV to predict early liver function decline. Curves illustrate the performance in distinguishing mALBI grade 1 from grades 2a or above (grade 2a, 2b, or 3). The diagonal line represents an AUC of 0.5 (no discriminatory ability)

The ROC curves for predicting early liver function decline (mALBI grade 1 vs. grades 2a or above) are shown in Fig. 5. The AUCs, optimal cutoff values, sensitivity, specificity, PPV, NPV, and accuracy are presented in Table 3. ROC analysis was performed to assess the ability of segmental fECV to distinguish early liver function decline (mALBI grade 1 vs. 2a or above). The fECV of segments S4, S5, and S8 demonstrated the highest diagnostic performance, with AUCs of 0.968 (95% CI: 0.925–0.990), 0.948 (95% CI: 0.899–0.978), and 0.932 (95% CI: 0.879–0.967), respectively (Table 3).

**Table 3:** Optimal cutoff values and diagnostic performance of hepatic fECV for each segment in predicting early liver function decline. (mALBI grade 1 vs. grades 2a or above)

	AUC (95% CI)	*p* value	Cut-off value	Sensitivity (%)	Specificity (%)	PPV (%)	NPV (%)	Accuracy (%)
S2 S3 S4	0.875 (0.810–0.924)	< 0.001	22.30	70.2	90.4	93.0	62.7	77.4
	0.899 (0.838–0.943)	< 0.001	21.33	69.1	94.2	95.6	62.8	78.1
	0.968 (0.925–0.990)	< 0.001	21.06	92.6	90.4	94.6	87.0	91.8
S5 S6 S7	0.948 (0.899–0.978)	< 0.001	21.19	87.2	86.5	92.1	78.9	87.0
	0.888 (0.825–0.934)	< 0.001	21.73	75.5	92.3	94.7	67.6	81.5
	0.895 (0.834–0.940)	< 0.001	19.98	81.9	84.6	90.6	72.1	82.9
S8	0.932 (0.879–0.967)	< 0.001	19.74	90.4	86.5	92.4	83.3	89.0

AUC-ROC = area under the receiver operating characteristic curve, CI = confidence interval, PPV = positive predictive value, NPV = negative predictive value

**Fig. 6:**
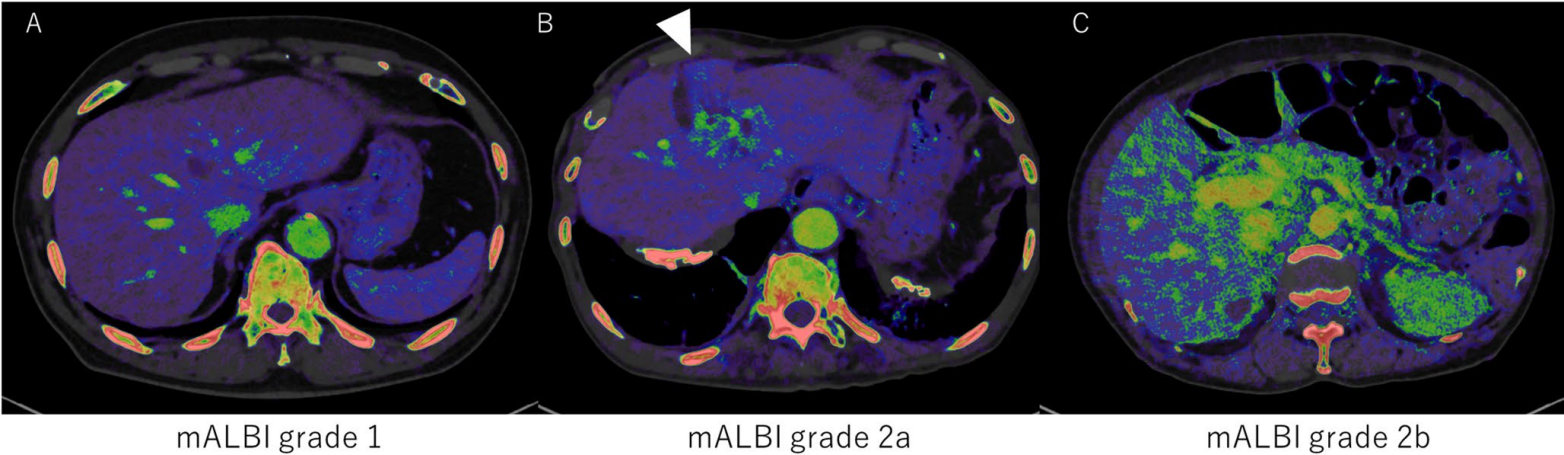
Representative color-coded fECV maps from patients with mALBI grades 1 (**A**), 2a (**B**), and 2b (**C**). fECV increases with liver function decline. In the grade 2a case (**B**), elevated fECV is ob-served in the medial segment (S4)

We present Fig. 6 to visually demonstrate representative color-coded fECV maps from patients with mALBI grades 1, 2a, and 2b (Fig. 6A–C). Notably, in the grade 2a case (Fig. 6B), a higher fECV is observed in the medial segment (S4), highlighting regional changes associated with early liver function decline.

## Discussion

This study demonstrated a strong correlation between fECV, calculated from 3-minute equilibrium phase PCD-CT images, and the mALBI grade, indicating its utility in assessing liver function. Furthermore, significant variations in fECV reflecting the mALBI grade were observed across different liver segments.

Previous studies using DECT have also shown a strong correlation between fECV and liver function or fibrosis stage [4–9]. The fECV represents both the extravascular extracellular space and the intravascular space. With fibrosis progression, collagen fiber synthesis expands the extravascular extracellular space. Since fibrosis primarily affects the extravascular compartment, increases in fECV associated with fibrosis are mainly driven by changes in this space. Normalization using hematocrit and aortic iodine density helps minimize the contribution of the intravascular space to the fECV calculation [5].

Prior research has indicated that fibrosis progression may be more pronounced in the medial segment (S4) of the left lobe and the anterior segments (S5, S8) of the right lobe compared to other segments [4]. Morphological changes associated with liver cirrhosis often involve atrophy of the right lobe (primarily S5–S8) and the medial segment (S4), accompanied by hypertrophy of the caudate lobe (S1) and the lateral segment (S2, S3) [22, 23]. As fibrosis progresses, it leads to an expansion of the extravascular extracellular space and a relative reduction of the intravascular space within the affected tissue. This suggests that fibrosis might progress earlier in regions prone to atrophy, leading to an earlier increase in fECV in these areas. To substantiate this hypothesis, future research is needed to quantitatively measure the volume of each segment and directly verify the correlation between the degree of atrophy and fECV values. Consistent with this hypothesis, our study found that in mALBI grade 2a, fECV was significantly higher in S4, S5, and S8 compared to other segments. In the more advanced mALBI grades 2b and 3, where fibrosis has likely progressed throughout the liver causing global functional decline, significant differences in fECV among segments were no longer observed. The lack of significant inter-segmental fECV differences in mALBI grades 2b and 3 is likely because fibrosis had progressed throughout the entire liver parenchyma, leading to a comprehensive decline in liver function that obscured the relative differences between segments. This suggests that fECV in specific segments may be a sensitive indicator for particular stages of liver function decline.

These findings highlight the importance of considering segmental variations in fECV relative to the mALBI grade. Notably, the fECV values of potentially atrophic areas—specifically the medial (S4) and anterior (S5, S8) segments—were significantly higher in mALBI grade 2a, demonstrating their usefulness in detecting early-stage liver function decline. ROC analysis further supported this, showing that S4, S5, and S8 yielded the highest AUCs (0.968, 0.948, and 0.932, respectively) for differentiating mALBI grade 1 from grade 2a or higher. This segmental pattern is also visually evident in representative cases, as shown in Fig. 6. In particular, the fECV map of the mALBI grade 2a patient (Fig. 6B) clearly demonstrates elevated values in the medial segment (S4), supporting the notion that early fibrosis-related functional decline may be regionally accentuated. These findings suggest that fECV in these regions may serve as sensitive biomarkers for early disease progression. In contrast, while the AUCs for the posterior segments (S6, S7) were relatively lower in early-stage prediction, only these segments exhibited statistically significant increases in fECV across all consecutive mALBI grades. This trend indicates that fECV in S6 and S7 may be better suited for evaluating the stepwise progression of hepatic functional impairment. However, we acknowledge that comprehensive stratification of liver disease severity may require whole-liver assessment, and further validation is needed to establish the prognostic value of segmental fECV. Taken together, our results imply that fECV provides complementary clinical value depending on the hepatic segment evaluated—serving both as an early detection tool (S4, S5, S8) and a staging marker for disease severity (S6, S7). The ability to examine fECV in each liver segment highlights an advantage of CT. Liver biopsy is limited to specific regions, and its invasiveness precludes multi-segment evaluation [24, 25]. Elastography techniques (ultrasound or MRI) are often restricted, primarily to the right lobe, due to technical challenges and artifact susceptibility, making comprehensive whole-liver assessment difficult [26, 27]. CT, however, offers advantages such as rapid acquisition, relative ease of performance, and reduced motion artifacts, enabling segmental fECV evaluation. It is important to position the clinical utility of segmental fECV not as a replacement for, but as a complement to established blood-based markers such as the mALBI grade. While mALBI provides a reliable and cost-effective measure of current liver function, fECV offers insight into the underlying structural pathology—namely fibrosis—that drives future, irreversible decline. Its potential lies in serving as an early predictive biomarker. Moreover, because contrast-enhanced CT is routinely performed for indications such as HCC surveillance, fECV can be calculated opportunistically without additional cost or radiation exposure. Unlike global blood tests, segmental fECV enables mapping of spatial heterogeneity, which may refine clinical decision-making—for example, by guiding biopsy to the most fibrotic regions or informing preoperative planning for hepatic resections and locoregional therapies.

Traditionally, fECV measurement required both non-contrast and equilibrium phase images, introducing potential misalignment issues; DECT largely resolved this problem [28, 29]. Conventional EID-based DECT systems utilize various approaches (dual-source, rapid kV-switching, dual-layer detectors) for spectral separation. However, these methods inherently involve significant overlap between the energy spectra, limiting the accuracy of energy discrimination [30]. PCD-CT, employing a single X-ray tube and a photon-counting detector, directly measures the energy of each incoming photon. This fundamentally different approach avoids energy spectra overlap. Consequently, PCD-CT offers improved energy discrimination performance compared to DECT, which is advantageous for accurate material decomposition, including iodine quantification [10–12]. A key practical advantage is that PCD-CT can acquire spectral data during routine scanning protocols, allowing retrospective spectral analysis whenever needed. Moreover, spectral analysis can be performed reliably even under high-speed or low-dose acquisition settings, which is especially advantageous for pediatric or critically ill patients.

Although studies on fECV measurement using PCD-CT are currently limited due to its recent clinical introduction, our study successfully performed detailed segmental fECV analysis using this technology. The correlation strength and stratification capability observed between PCD-CT-derived fECV and mALBI grade in our study were comparable to those reported in previous DECT studies [4]. While specific imaging parameters and equilibrium phase timing differed from prior research, the accuracy of hepatic iodine quantification in the equilibrium phase appears similar between DECT and PCD-CT. However, PCD-CT provides notable workflow benefits, facilitating straightforward fECV calculation within routine clinical practice. Additionally, the inherent lower noise characteristics of PCD-CT, particularly relevant at potentially lower doses used in clinical practice, likely contribute to the accurate calculation of fECV. While a prior study by Ozaki et al. demonstrated segmental fECV differences in chronic liver disease using 5-minute delayed DECT [4], our study expands this framework by incorporating PCD-CT with a shorter (3-minute) equilibrium phase. This approach not only reflects practical imaging work-flows but also enables retrospective spectral reconstruction, allowing flexible application without protocol modification. Moreover, by investigating the prognostic impact of segment-specific fECV on future mALBI grade deterioration, our study provides novel insight that was not addressed in previous DECT-based evaluations.

Our choice of a 3-minute delay for the equilibrium phase imaging warrants justification, as it is shorter than the 5 to 10-minute delay used in many other DECT-based fECV studies. Our rationale was threefold. First, our primary goal was to validate a protocol that aligns with our institution’s routine dynamic liver CT imaging, thereby maximizing the potential for direct clinical translation and future retrospective analyses. Second, while the optimal timing remains debated [4–9], key studies have reported no significant difference in the correlation with fibrosis grade between 3-minute and 5-minute or 10-minute images [7, 31], suggesting that a 3-minute delay is sufficient to achieve contrast equilibrium. Third, our own results internally validate this approach, as the fECV derived from 3-minute images showed a strong correlation with mALBI grade and excellent diagnostic performance. While our retrospective design did not allow for a direct comparison with later time points, these factors combined support that our 3-minute protocol is both practical and effective for routine clinical application.

The high diagnostic performance of segmental fECV, particularly in segments S4, S5, and S8, for detecting early-stage (mALBI grade 2a) liver function decline has several potential clinical implications. First, it could refine prognostication and surveillance strategies. Patients with elevated fECV in these specific segments, even with otherwise reassuring global liver function tests, could be identified as a higher-risk population. This would allow for a more personalized surveillance schedule, with more frequent follow-up including elastography or blood markers, to detect progression to cirrhosis or hepatocellular carcinoma earlier. Second, segmental fECV could aid in treatment stratification. It might help identify candidates who would benefit most from early initiation of antifibrotic therapies or more aggressive management of the underlying etiology. From a clinical workflow perspective, segmental fECV could be implemented as an opportunistic screening tool. Since many patients with chronic liver disease undergo abdominal CT for various reasons, a protocol including a 3-minute equilibrium phase could provide valuable functional information without requiring a separate examination. The results could be presented as a quantitative ‘fECV map,’ offering an intuitive visual assessment of hepatic heterogeneity that complements the standard anatomical report. However, these potential applications are currently speculative and require validation in future prospective studies to confirm their clinical utility and impact on patient outcomes.

This study has several limitations. First, as a retrospective, single-center study, the generalizability of our findings is inherently limited. The results may be subject to selection bias and influenced by our institution’s specific patient demographics and clinical protocols. Furthermore, while the total cohort consisted of 146 patients, the subgroup of patients with the most severe liver impairment (mALBI grade 3) was small, with only 14 individuals. This limited sample size reduces the statistical power for this subgroup and may affect the robustness of our conclusions regarding advanced-stage liver disease. Therefore, our findings should be considered preliminary, and their validity needs to be confirmed by larger, multi-center prospective studies to ensure broader applicability. Second, we used the mALBI grade as the reference standard for liver function decline instead of liver biopsy, which is the gold standard for fibrosis assessment, consistent with some previous studies [4]. While liver biopsy is the gold standard for fibrosis assessment, our primary objective was to evaluate the utility of segmental fECV in predicting liver function, for which the mALBI grade is a well-validated and clinically relevant marker. This choice was also practical, reflecting clinical scenarios where an invasive procedure like biopsy is not always feasible, especially in a retrospective setting. Nonetheless, this represents a significant limitation. The fECV is believed to directly reflect the expansion of the extracellular matrix, which is a key histological feature of fibrosis. In contrast, the mALBI grade is a surrogate marker that primarily reflects liver function through albumin and bilirubin levels. Although liver function and the degree of fibrosis are closely related, they are not interchangeable. A discordance can exist where significant fibrosis is present with preserved liver function, or vice versa. Therefore, our findings demonstrate a correlation between segmental fECV and a functional score, but the direct relationship with the underlying histologic stage of fibrosis requires further investigation. Future prospective studies that include direct comparison with liver histology or other established non-invasive fibrosis markers, such as elastography, are necessary to validate the utility of segmental fECV in accurately staging fibrosis and predicting clinical outcomes. Furthermore, while a subset of our cohort had undergone liver biopsy, the significant temporal gap between the biopsy and the CT scan, along with non-standardized histological reporting, precluded a meaningful correlation analysis. A direct comparison with contemporaneous histological data remains a critical goal for future research. Third, this study has several limitations regarding potential confounding factors. Our study population was heterogeneous, including patients with chronic liver disease of various etiologies (e.g., viral, alcoholic, and metabolic). We did not perform a subgroup analysis based on etiology, as the number of patients in each category was insufficient for a statistically meaningful comparison. This heterogeneity is a significant limitation, as the underlying pathophysiology of fibrosis can differ between etiologies and may contribute to variability in fECV. Furthermore, we did not systematically account for other important confounders known to influence fECV values, such as hepatic steatosis, iron overload, active hepatitis, and ascites. These conditions can alter tissue density, vascular permeability, and contrast agent distribution, thereby affecting the accuracy of fECV measurements. For instance, severe steatosis may reduce the baseline attenuation of the liver parenchyma, while active inflammation could increase extracellular fluid. Due to the retrospective nature of our study, a systematic analysis of these factors was not feasible, and their potential influence on our findings cannot be excluded. Future prospective studies are therefore essential; they should incorporate these variables and utilize combined imaging techniques, biochemical markers, or histopathologic validation to better isolate the impact of each confounder and refine the diagnostic utility of segmental fECV. Fourth, fECV values were normalized to aortic iodine density, which may vary with cardiac output and systemic circulation. Although this method is widely used in previous studies [4–9] and offers practical reproducibility, it may introduce confounding. We did not perform a sensitivity analysis to evaluate the impact of circulatory variability. Future studies should consider alternative normalization strategies or include sensitivity analyses to further validate fECV robustness. A further limitation is that ROI placement for fECV measurement remains operator-dependent. Although we reported good to excellent interobserver agreement, intra-observer reproducibility was not assessed. This may affect the consistency of measurements, particularly in clinical settings. Future studies should evaluate intra-observer variability to further establish the reliability of segmental fECV quantification. Finally, it is important to frame this study as a proof of concept. While our findings are promising, they remain exploratory due to several limitations. As discussed, we did not systematically account for all potential confounders, and our reference standard—the mALBI grade—is a functional surrogate rather than a direct measure of fibrosis. Moreover, the optimal cut-off values derived from ROC analysis were not validated using external datasets, nor did we perform internal validation techniques such as cross-validation. These thresholds should therefore be considered hypothesis-generating. To substantiate the clinical utility of segmental fECV, future prospective studies are needed. Such studies should include larger and more diverse patient populations, incorporate established non-invasive fibrosis markers such as MR elastography, and evaluate clinical outcomes to comprehensively validate this approach.

## Conclusion

Segmental fECV measurement using a 3-minute equilibrium phase PCD-CT offers preliminary insights into liver function assessment in chronic liver disease. In our cohort, fECV in the medial (S4) and anterior (S5, S8) segments—particularly elevated in mALBI grade 2a—showed potential as an early indicator of functional decline, while fECV in the posterior segments (S6, S7) increased progressively across mALBI grades, suggesting utility in disease staging. This segment-specific approach may support individualized disease monitoring and treatment planning. While our study confirms the feasibility of deriving segmental fECV from a routine clinical protocol and highlights the technical advantages of PCD-CT for quantitative analysis, we acknowledge that the clinical application of this technique remains limited. The widespread availability of PCD-CT scanners and dedicated fECV analysis software is not yet established. Therefore, although our findings suggest potential utility, further prospective validation—including outcome-based studies—is necessary before segmental fECV can be broadly adopted in clinical practice.

## Data Availability

The files/data used to support the findings of this study are available from the corresponding author upon request.
